# Principled Approaches to Direct Brain Stimulation for Cognitive Enhancement

**DOI:** 10.3389/fnins.2017.00650

**Published:** 2017-11-30

**Authors:** Vishnu Sreekumar, John H. Wittig, Timothy C. Sheehan, Kareem A. Zaghloul

**Affiliations:** Surgical Neurology Branch, National Institute of Neurological Disorders and Stroke, National Institutes of Health, Bethesda, MD, United States

**Keywords:** invasive brain stimulation, brain-computer interface, intracortical, memory, higher cognition

## Abstract

In this brief review, we identify key areas of research that inform a systematic and targeted approach for invasive brain stimulation with the goal of modulating higher cognitive functions such as memory. We outline several specific challenges that must be successfully navigated in order to achieve this goal. Specifically, using direct brain stimulation to support memory requires demonstrating that (1) there are reliable neural patterns corresponding to different events and memory states, (2) stimulation can be used to induce these target activity patterns, and (3) inducing such patterns modulates memory in the expected directions. Invasive stimulation studies typically have not taken into account intrinsic brain states and dynamics, nor have they a priori targeted specific neural patterns that have previously been identified as playing an important role in memory. Moreover, the effects of stimulation on neural activity are poorly understood and are sensitive to multiple factors including the specific stimulation parameters, the processing state of the brain at the time of stimulation, and neuroanatomy of the stimulated region. As a result, several studies have reported conflicting results regarding the use of direct stimulation for memory modulation. Here, we review the latest findings relevant to these issues and discuss how we can gain better control over the effects of direct brain stimulation for modulating human memory and cognition.

## 1. Introduction

Major advances have been made recently in the use of intracortical brain-computer interfaces (iBCI) to restore movements in patients with disorders such as tetraplegia (Bouton et al., 2016; Ajiboye et al., 2017). A similar approach was employed successfully to implement autonomous and open-ended communication in a patient with amyotrophic lateral sclerosis (ALS) who, prior to the use of the iBCI, was restricted to communicating using eye movements or blinks in response to close-ended questions (Vansteensel et al., 2016). In this application, decoded electrocorticographic patterns corresponding to movement intentions were fed into a computer system that directed typing software. The success of these motor cortex iBCIs relies on the quality of decoded neural signals related to different movement intentions and the ability to then control muscular activity via a neuromuscular electrical stimulation system. The movement-based applications discussed above simply decode normal neural activity but do not actively modify brain activity itself. However, in a higher cognitive application, which is the focus of our review here, the desired output is a brain state and not a movement pattern.

To understand the different challenges involved in developing an iBCI for supporting higher cognition during unconstrained naturalistic experience, consider a hypothetical device that improves memory in patients with a memory disorder. One approach could be to have the patients use a wearable device that continuously captures what they see and hear (Sreekumar et al., 2014, 2017; Nielson et al., 2015). Machine learning algorithms could then be applied to determine the mappings between experience and neural activity that support better memory formation and those that do not. These algorithms will need to be dynamically updated since both experience and neural processing can change over time. Ultimately, after training the algorithms, this iBCI must be able to detect a “bad” environment-neural mapping (possibly also given a readout of intended thought or action) and provide cognitive support by inducing desirable neural activation patterns in the brain. In severe cases of brain damage, a neural prosthesis has to substitute the function of one or more brain regions. Even though this is a more challenging prospect, a method involving mapping neural activity patterns in one region to those in another (often in a nonlinear manner) and then artificially inducing the desired response in the target brain region via invasive brain stimulation has shown promise as a hippocampal prosthesis in rats (Berger et al., 2012). Whether such mappings can be learned using data from healthy animals and then be generalized to treat disorders in other animals of the same species is a question that remains to be fully addressed. Such a generalization would be possible only if there are across-subject generic patterns of task-specific processing.

Before the futuristic scenario described above can be a reality, we need to gain better control over the effects of stimulation within the laboratory. Therefore, in order to build an iBCI for supporting or enhancing cognitive function, we must not only be able to identify potential target brain states and induce such states via brain stimulation, but we must also establish that these “desirable” brain states have a causal role in modulating higher cognitive function, rather than merely being a correlate of some other underlying process. We organize our review around studies that have tackled these issues in the domain of human memory. Specifically: (1) Are there reliable neural patterns corresponding to different events and memory states? (2) Can these neural activity patterns be induced in the brain via stimulation? (3) Does inducing these patterns using stimulation causally modulate memory in the expected directions?

## 2. Are there reliable neural patterns corresponding to different events and memory states?

Studies using different modalities of brain imaging and electrophysiology have shown that individual items/events can be represented differently at multiple scales ranging from distributed patterns across the whole brain to highly localized spiking activity patterns and that the corresponding neural representations are reinstated when successfully remembering those items (Johnson and Rugg, 2007; Manning et al., 2011; Deuker et al., 2013; Yaffe et al., 2014; Jang et al., 2017). The fact that successful memory of an event entails reinstatement of the specific neural activity present during encoding of that event implies that it should be possible to decode the contents of these neural activity patterns. Most attempts to date have decoded memory contents at the level of category in part due to the limited amount of time available in a laboratory setting to obtain a sufficient number of repetitions of each individual item to train a classifier (Naselaris et al., 2011). However, voxel-wise modeling of fMRI data to map responses across the human cortex to different words as people listened to several hours of narrative stories has revealed that semantic maps are highly consistent across participants and that different categories are represented in different brain regions (Huth et al., 2016).

One major limitation to the human studies reviewed above is that they are restricted to studying relatively short spatiotemporal scales. Monitoring posterior parietal cortex (PCC) neurons for a period of a month as mice performed a virtual-navigation task revealed that the correspondence between individual cells' activity and task features were stable over timescales of a day but underwent major reorganization across days and weeks (Driscoll et al., 2017). However, the information that could be decoded from population activity remained relatively stable for over a week despite reorganization at the level of individual neurons and the development of new representations as mice learned new associations did not greatly perturb existing representations. Longer term human studies are necessary to establish how stable representations are over time which will help determine how often to update algorithms in iBCIs that connect brain states to experience. Such studies can be performed in patients with electrode implants undergoing continuous presurgical epilepsy monitoring for several days or weeks.

At a coarser level, general memory-related changes in oscillatory neural activity have been observed in multiple studies. Neural oscillatory changes accompanying successful memory formation relative to unsuccessful encoding, i.e., the subsequent memory effect (SME; Paller and Wagner, 2002), have been found in different frequency bands, with most studies reporting a general decrease in low frequency power and increase in high frequency power during successful encoding (Sederberg et al., 2006; Guderian et al., 2009; Fell et al., 2011; Long et al., 2014; but see Hanslmayr and Staudigl, 2014 on the sensitivity of SMEs on encoding task and contextual overlap between study and test). Successful memory formation is also accompanied by flatter power spectral density (PSD) slopes and increases in sample entropy (Sheehan et al., 2017), both measures of signal complexity, suggesting that the ability to successfully encode information in indexed by neural signal complexity. Finally, improved functional interactions between brain regions such as the MTL and PFC may underlie successful memory formation (Fell et al., 2003). If these oscillatory activity patterns and coherent interactions between brain regions have a causal role in memory formation and retrieval, then successful modulation of memory performance could potentially be achieved by stimulation that induces these specific patterns in the brain.

## 3. Can specific neural activity patterns be induced in the brain via stimulation?

If we are to leverage what we know about naturally occurring neural patterns that accompany successful memory formation to gain control over effects of stimulation, it may be desirable to apply stimulation in such a way that it respects intrinsic brain states and dynamics. This is particularly important when we want to use stimulation to attribute causation to the naturally occurring memory-related neural patterns observed in previous studies in the absence of stimulation (Jazayeri and Afraz, 2017). Advanced optogenetics experiments, for this reason, target specific pathways in order to attain perturbations that stay within the neural manifold of interest (Gradinaru et al., 2010; Yizhar et al., 2011; Janak and Tye, 2015; Jazayeri and Afraz, 2017). Some electrical stimulation studies have also employed a similar approach by targeting structures that have direct connections to the hippocampus (e.g., Koubeissi et al., 2013) which led to improvements in memory, whereas most studies that directly stimulated the hippocampus reported decreased memory performance (see Kim et al., 2016 for a review). One important consequence of stimulation producing unnatural states and dynamics is that null effects become less informative because abnormal perturbations failing to influence behavior does not provide diagnostic evidence for the presence or absence of a neural code for the behavior under investigation in the target region (Jazayeri and Afraz, 2017).

Perhaps one reason that direct brain stimulation to enhance human memory has met with mixed success is that it has not been used to specifically target previously identified memory-related patterns of activity. Typically, random stimulation is applied without considering a priori if the effects of that stimulation recapitulate the patterns previously identified as underlying successful memory processes. For instance, if oscillatory mechanisms indeed have a causal role in cognition, then the successful application of stimulation may depend on its ability to entrain specific oscillations. Transcranial alternating current stimulation (tACS) at 10 Hz increases parieto-occipital alpha activity and in turn modulates target detection performance in a visual oddball task (Helfrich et al., 2014; Mierau et al., 2017) suggesting a causal role for alpha oscillations. Conversely, the failure to entrain oscillations leads to a failure to modulate neural excitability and cognitive performance (Braun et al., 2017). Recently, it was demonstrated that high frequency bursts of direct brain stimulation in humans result in local entrainment of oscillations (Amengual et al., 2017), suggesting ways in which invasive stimulation can be used to induce specific patterns of activity thought to underlie successful memory processes.

Other mechanisms like synchronization of activity in different brain regions may also have important roles in modulating cognitive performance. For example, theta synchronization between the MTL and frontal regions relates to successful memory formation (Fell et al., 2003; Anderson et al., 2010; Burke et al., 2013) and the causal role of such synchronization can be investigated using stimulation to modulate connectivity (e.g., Wang et al., 2014; Johnen et al., 2015; Nilakantan et al., 2017). In a recent comprehensive review of invasive and non-invasive stimulation studies of memory modulation, Kim et al. (2016) focused on such a network perspective, arguing for a stimulation approach that targets interactions between brain regions underlying memory rather than targeting isolated brain regions. A recent study demonstrated the feasibility of characterizing the effects of local direct brain stimulation on the rest of the brain using simultaneous electrical stimulation and fMRI in humans (Oya et al., 2017) which may pave the way to designing invasive stimulation studies that target memory-relevant interactions between brain regions (also see Muldoon et al., 2016 for a computational modeling approach to explore system-wide impacts of targeted stimulation). Similarly, Shine et al. (2017) determined participants' resting state networks using fMRI and then applied invasive electrical stimulation to nodes within the default (DN), frontoparietal (FPN), and salience networks (SN) to understand the precise spatiotemporal dynamics underlying directed interactions between brain regions that support memory and cognition.

Finally, network control theory can be used to determine if particular nodes or sets of nodes in a brain network can be manipulated in order to drive the system into specific sequences of activity states that may be relevant for cognition. Gu et al. (2015) employed different controllability measures to identify brain areas that drive different control goals and found that *average controllability*, capturing the ability to steer the system into different states with little effort, was greatest in default mode network hubs; *modal controllability*, identifying the ability to drive the system into difficult-to-reach states, was greatest in canonical cognitive-control networks such as the frontoparietal and cingulo-opercular systems; and *boundary controllability*, capturing the ability to integrate information across different cognitive processes, was distributed across systems but particularly in the ventral and dorsal attention systems. Utilizing such tools to select nodes in order to achieve specific control strategies may be beneficial in designing targeted stimulation studies.

## 4. Effects of direct brain stimulation on memory

Here, we briefly review recent invasive stimulation studies of memory, most of which did not a priori target precise neural activity patterns. Therefore, it is difficult to make causal inferences about previously identified neural processes that accompany successful memory encoding and retrieval in the absence of stimulation. However, existing stimulation studies may provide relevant information regarding other factors such as the time of stimulation, brain state, and how these factors influence stimulation effects. Furthermore, *post-hoc* analyses may help determine whether random stimulation leading to improvement in memory was accompanied by neural activity previously identified as playing an important role in successful memory processes.

Demonstrating the influence of time of stimulation at a coarse scale, Merkow et al. (2015) found that stimulating during the distractor period between encoding and recall had a more negative impact on recall performance compared to stimulating during either encoding or retrieval. Furthermore, stimulation during the distractor period also reduced the typical tendency for people to begin recall of short lists with the first item. These results suggested that stimulation either interferes with rehearsal during the distractor period or that it disrupts the normal neural processes underlying temporal context drift and therefore may impact people's ability to use temporal context as a retrieval cue during recall. More precise temporal effects of stimulation were reported by Ezzyat et al. (2017) who trained a logistic regression classifier to predict memory formation success in a free recall task based on spectral power features. The classifier relied on low-frequency power decreases and simultaneous high-frequency power increases across frontal, temporal, and occipital cortices to predict successful recall, recapitulating results from prior SME studies. Critically, Ezzyat et al. (2017) found that direct stimulation delivered during a “bad” encoding state as indicated by the classifier output improved both recall performance as well as classifier evidence for good encoding whereas stimulation applied during a “good” encoding state decreased both recall performance and classifier evidence. These results suggest that the effect of stimulation on brain function depends on the state of neural activity at the time of application of stimulation and further demonstrate the possibility that specifically targeting neural patterns identified in prior SME studies may yield more reliable improvements in memory.

Invasive brain stimulation studies have reported both positive and negative effects on memory in humans. For example, Suthana et al. (2012) reported positive effects of direct stimulation of the entorhinal cortex on spatial memory. However, Jacobs et al. (2016), in an analysis of a larger cohort of patients, reported a deleterious effect of stimulation in both the entorhinal region and the hippocampus on both spatial and verbal memory. The two studies employed different spatial memory tasks that may have recruited different processes. Additionally, stimulation was applied for 10 s per trial in Jacobs et al. (2016) whereas Suthana et al. (2012) applied stimulation for much longer durations. It is also possible that the two studies targeted different areas within the entorhinal cortex and stimulation effects are sensitive to the specific brain region being stimulated. For example, electrically stimulating a specific hotspot within the monkey perirhinal cortex with many stimulus-selective neurons made the monkeys endorse an item encountered previously as familiar (OLD) but stimulating a fringe region of that same hotspot produced the opposite effect where monkeys falsely rejected a previously seen item (NEW) (Tamura et al., 2017). Based on the neuroanatomy of the perirhinal cortex which is characterized by short-range inhibitory connections to neighboring neurons and longer collateral projections to adjacent regions, Tamura et al. (2017) posited that electrical stimulation of the hotspot inhibited the neighboring regions which had little impact on task performance whereas stimulating the peripheral regions inhibited activity in the hotspot with stimulus-specific neurons which impacted performance (inducing a NEW bias). Therefore, small differences in the specific neuronal populations within the entorhinal cortex that Jacobs et al. (2016) and Suthana et al. (2012) targeted may similarly have led to inconsistent effects. Indeed, in the latest human entorhinal direct brain stimulation study, a refined approach by using physiologic level currents to deliver precise stimulation via microelectrodes to the right entorhinal area containing afferent inputs to the hippocampus was successful in enhancing recognition memory for photographs (Titiz et al., 2017).

Logothetis et al. (2010) used simultaneous electrical stimulation, electrophysiology, and fMRI to show that stimulation can cause both decreases and increases in downstream brain activity depending on the stimulation site and frequency of stimulation. The optimal target neural activity may also be highly dependent on brain region and task. For example, decreasing visual cortex excitability increases strength of imagery whereas the opposite is true of the prefrontal cortex (Keogh et al., 2016). This dependence of stimulation effects on brain region is further supported by the finding that tactile synchronous costimulation of two digits on the hand at, above, or below the resonance frequency of the somatosensory cortex selectively affects functional topography and connectivity (Lea-Carnall et al., 2017). These results demonstrated that plasticity in the human cortex is modulated by both stimulation frequency and its relationship with the resonance frequency of the cortical region under investigation. On a related note, different brain regions have different intrinsic processing timescales (Lerner et al., 2011; Honey et al., 2012) and Cocchi et al. (2016) used non-invasive cortical stimulation in humans and demonstrated that inhibiting a region can cause increased functional interactions with another region if stimulation causes the timescales of their activity to match and decreased communication if stimulation produces a greater mismatch in these timescales. Therefore, direct brain stimulation parameters that respect the intrinsic properties of the target brain region(s) may be more effective in generating activity as well as in promoting communication with other regions relevant for memory. State-dependence of induced effects has been relatively well explored in non-invasive domains like transcranial magnetic stimulation (TMS) (Silvanto et al., 2008) and cohesive theoretical frameworks have been proposed to account for facilitations and disruptions under different task conditions that promote different levels of neuronal excitability (Silvanto and Cattaneo, 2017). A similar principled approach to direct brain stimulation has been lacking.

Furthermore, invasive brain stimulation is highly localized relative to non-invasive methods, and given the heterogeneity of neural responses at small scales, it is imperative that we understand more precisely the relationship between stimulation parameters and the response elicited from a small piece of neural tissue (e.g., Winawer and Parvizi, 2016) as any small differences in those “initial conditions” (e.g. location, stimulation parameters, existing state of the tissue) can lead to substantial differences in the elicited response given the complexity of the system we seek to perturb. Therefore, given the many factors that potentially influence stimulation effects, it is unsurprising perhaps that effects of stimulation on memory are highly variable both across studies as well as across individuals within studies (Halgren and Wilson, 1985; Perrine et al., 1994; Coleshill et al., 2004; Lacruz et al., 2010; Suthana et al., 2012; Fell et al., 2013; Hanslmayr et al., 2014; Miller et al., 2015; Jacobs et al., 2016; Ezzyat et al., 2017; also see Kim et al., 2016 for a recent review).

## 5. Discussion: toward a higher cognitive brain-computer interface

In this review, we identified several factors that potentially influence the effects of brain stimulation on cognition. As Braun et al. (2017) recently argued in the domain of tACS, we need modeling studies of how these factors relate to stimulation effects and extensive experimental validation of the models. Ultimately, task demands, the brain region being stimulated and its intrinsic properties, the current dynamic state, time and duration of delivery of stimulation and the specific stimulation parameters all interact to determine the cognitive and behavioral consequences of stimulation (Silvanto et al., 2008; Romei et al., 2016). We outlined a more principled approach to designing studies in order to gain better control over how invasive brain stimulation modulates human memory and cognition. Such an approach targets specific neural activity patterns previously determined to underlie successful memory processing, and utilizes tools from domains such as control theory to predict the efficacy of manipulating specific nodes within the network to effect brain states. Only using such a principled approach can we gain a sufficiently detailed understanding of how direct brain stimulation modulates human memory and cognition, in order to build an intracortical BCI for cognitive enhancement.

## Author contributions

VS drafted the manuscript. JW, TS, and KZ provided critical comments and edits.

### Conflict of interest statement

The authors declare that the research was conducted in the absence of any commercial or financial relationships that could be construed as a potential conflict of interest.
